# Knockdown of long non-coding RNA HOTAIR promotes bone marrow mesenchymal stem cell differentiation by sponging microRNA miR-378g that inhibits nicotinamide N-methyltransferase

**DOI:** 10.1080/21655979.2021.2006863

**Published:** 2021-12-13

**Authors:** Wei Wang, Tao Li, Shibo Feng

**Affiliations:** Department of Orthopedics, WuHan HanKou Hospital, Wuhan, Hubei, China

**Keywords:** HOTAIR, miR-378g, differentiation, osteoporosis, osteogenic

## Abstract

Osteoporosis (OP) is associated with a serious social and economic burden. Recent studies have shown that the differential expression of long non-coding RNAs (lncRNAs) is closely related to OP. However, the specific molecular mechanism of HOX transcript antisense intergenic RNA (HOTAIR) remains to be elucidated.

The expression of HOTAIR and miR-378g in OP patients was detected using quantitative reverse transcription polymerase chain reaction (qRT-PCR). Bone marrow mesenchymal stem cells (BMSCs) were isolated and cultured, and osteogenic differentiation was induced. Alkaline phosphatase (ALP) and Runt-related transcription factor 2 (RUNX2) were detected by qRT-PCR, ELISA, and Western blotting. Calcium deposition was measured using Alizarin red s (ARS) staining. Molecular interactions between HOTAIR, miR-378g, and nicotinamide N-methyltransferase (NNMT) were detected using a dual-luciferase reporter assay.

HOTAIR expression was upregulated and miR-378g level was downregulated in OP patients. HOTAIR expression decreased during the osteogenic differentiation of BMSCs. Silencing HOTAIR or NNMT reduced ALP and RUNX2 levels and promoted calcium deposition. The overexpression of HOTAIR or interference with miR-378g inhibited the osteogenic differentiation of BMSCs. HOTAIR negatively regulates miR-378g by targeting NNMT.

HOTAIR is an miR-378g sponge that targets NNMT, inhibits the osteogenic differentiation of BMSCs, and provides a valuable target for the treatment of OP.

## Introduction

Osteoporosis (OP), a bone metabolic disease caused by low bone mass and structural damage, has become a global public health problem [1,2]. OP has seriously affected the health and quality of life of patients and increases the economic burden of countries and families [3,4]. Damage to osteoblasts has been reported to be one of the factors leading to the occurrence and development of OP, and bone marrow mesenchymal stem cells (BMSCs) have been proven to have the ability to differentiate into osteoblasts and exert a certain mitigating effect on OP [5,6]. Therefore, it is of clinical value to improve the osteogenic differentiation ability of BMSCs to alleviate OP.

Long non-coding RNAs (lncRNAs) are non-coding transcripts that typically exceed 200 nucleotides in length and are one of the largest and most significantly diverse RNA families that have emerged in recent years [7]. LncRNAs are ubiquitous in many species. In fact, lncRNAs regulate gene expression at the epigenetic, transcriptional, and post-transcriptional levels, and participate in various biological processes [8–10]. LncRNAs have become an intriguing area of research and are widely studied in the diagnosis and treatment of various diseases [11]. Recent reports suggest that lncRNAs play key roles in bone development and disease [12]. For example, the expression of lncRNA-OG is upregulated during osteogenic differentiation and promotes the osteogenic development of BMSCs [13]. The lncRNA, BCAR4, participates in the osteogenic differentiation of BMSCs and exacerbates the progression of OP [14]. HOTAIR is one of the most widely studied maladjusted lncRNAs found in human cancer and has been shown to play a role in pathogenesis, disease progression, drug resistance, and reduced survival [15]. Moreover, the lncRNA, HOTAIR, has been shown to be overexpressed in the serum of patients with OP and inhibits the osteoblastic differentiation of BMSCs in rats [16]. However, the underlying mechanism of HOTAIR in OP remains to be explored.

MicroRNAs (miRNAs) are a class of non-coding RNAs (~22 nucleotides) that play a central role in the post-transcriptional regulation of protein-coding genes through mRNA cleavage, direct translation inhibition, and/or mRNA instability [17]. miRNAs have been reported to participate in the mechanisms of osteoblasts and osteoclasts related to OP [18,19]. For example, miR-335-5p induced the osteogenic differentiation and bone formation of BMSCs in mice and supported the potential application of BMSCs in craniofacial bone regeneration [20]. miR-34a targets NOTCH1 for the healing of tibial defects in irradiated rats and enhanced the osteogenic differentiation of BMSCs [21]. miRNA-378 has been reported to enhance osteogenic differentiation by regulating GalNT7 [22]. Moreover, miR-378g was found to be maladjusted in femoral head necrosis [23]. Nicotinamide N-methyltransferase (NNMT) is an important metabolic enzyme that is overexpressed in human diseases, including Parkinson’s disease, cardiovascular disease, cancer, and metabolic disorders [24]. NNMT has been found to be essential for the osteogenic differentiation of BMSCs. Further, NNMT participates in the osteogenic differentiation and bone formation of stem cells, and is upregulated in ovariectomized mice [25,26]. However, the effect of miR-378g/NNMT on osteogenic differentiation and OP has rarely been reported.

We hypothesized that the osteoblastic differentiation of BMSCs could be induced via the regulation of the lncRNA HOTAIR/miR-378g/NNMT signaling pathway to alleviate OP. Functional and mechanistic analysis of BMSCs revealed that HOTAIR upregulated the expression of NNMT through sponge miR-378g, and then inhibited osteogenic differentiation by reducing osteogenic differentiation-related factors, including ALP, alizarin red staining, and RUNX2. The purpose of this study was to determine the effect of HOTAIR/miR-378g/NNMT on the osteogenic differentiation of BMSCs to provide a research basis for the treatment of OP.

## Methods

### Clinical samples

Thirty patients with osteoporosis and 30 patients with non-osteoporotic fractures who were admitted to our hospital were enrolled in this study. The participants fasted for 8 h after admission, and 5 mL of blood was intravenously extracted from each patient. After blood coagulation, the supernatant was centrifuged at 1500 g to separate the serum, which was stored for later use. This study was approved by the Ethics Committee of our hospital, and written consent was obtained from all participants prior to blood collection.

### Isolation, culture, and differentiation of BMSCs

The animal experiment plan was reviewed and approved by the Animal Care and Use Committee of our hospital. All rats were kept in a specific pathogen-free animal room in the experimental animal research center of our hospital. BMSCs were isolated from the tibia and femur of 10-week-old Sprague-Dawley female rats (weight, 50–60 g) purchased from Hunan SJA Laboratory Animal (China). Briefly, the bone marrow was washed with phosphate buffered saline (PBS) and cultured in α-MEM (Invitrogen, USA) supplemented with 10% fetal bovine serum (FBS), 1% penicillin/streptomycin, and 2-mercaptoethanol (2-ME) at 37°C in an incubator containing 5% CO_2_. The culture medium was changed every 3 days to remove free cells. After 5 days, the primary cells were trypsinized and subcultured [27]. When the convergence of the third generation reached 70–80%, the osteogenic differentiation of BMSCs was induced using differentiation medium containing α-MEM, 10% FBS, 100 μg/mL ascorbic acid, 2 mM β-glycerophosphate, and 10 nM dexamethasone [28].

### Alkaline phosphatase (ALP) activity assay

ALP activity was measured by converting colorless p-nitrophenyl phosphate (pNPP, Sigma, USA) into colored p-nitrophenol. Briefly, after washing twice with PBS, the cells were scraped into a solution containing 20 mM Tris-HCl (pH 8.0), 150 mM NaCl, 2% Triton X-100,0.02% NAN3, and 1 μg/mL aprotinin. Thereafter, ALP activity at 405 nm was measured with pNPP as a substrate on a spectrophotometer (Agilent Technologies, USA) [29].

### Alizarin red S (ARS) staining

The matrix of the BMSCs was evaluated after osteoinduction. Briefly, BMSCs were incubated in 70% ethanol at 25°C for 1 h and then stained with 40 mM ARS for 10 min. The stained BMSCs were photographed using a light microscope (Olympus, Tokyo, Japan) [30].

### Quantitative reverse transcription polymerase chain reaction (qRT-PCR)

Total RNA was extracted from blood and cells using the Norgen Biotek Total RNA Purification Kit (Fermentas, USA). RNA was reverse-transcribed using the RevertAid First Strand cDNA Synthesis Kit (Fermentas). qRT-PCR of mRNA was performed on a Bio-Rad iQ5 thermal cycler (Bio-Rad, USA) using SYBR Green Supermix (Bio-Rad). Glyceraldehyde-3-phosphate dehydrogenase (GAPDH) was used as control for HOTAIR, NNMT, ALP, and RUNX2.

The miRNAs in serum samples were collected using the Qiagen miRNeasy Serum/Plasma Kit (Qiagen, Germany). The obtained RNA mixture was eluted in 20 μL RNase-free water and stored at −80C. Isolation of miRNAs in BMSCs was performed using the mirVana miRNA isolation kit (Ambion, USA). Reverse transcription of miRNA was performed using Ncode miRNA first-strand cDNA synthesis kits (Invitrogen). qRT-PCR was performed on a 7900HT Fast Real-Time System (Applied Biosystems, USA). U6 was used as an endogenous control for miR-378g. The relative expression of each miRNA was determined using the 2^−ΔΔCT^ method. The primer sequences are listed in Table 1.

**Table 1.** The human primer sequences used for Real-Time PCR

Gene	Primer sequence
HOTAIR	Forward: 5′-GGGTGTTGGTCTGTGGAACT-3′
	Reverse: 5′-CAGTGG-GGAACTCTGACTCG-3′
miR-378g	Forward:5′-ACACTCCAGCTGGGGAAGACTGAGGTTC-3′
	Reverse:5′-CTCAACTGGTGTCGTGGAGTCGGCAATTCAGTTGAGAGCCCAGT-3′
NNMT	Forward: 5′-AGGAACCAGGAGCCTTTGACT-3′
	Revers: 5′-CCTGAGGGCAGTGCGATAGG-3
RUNX2	Forward:5ʹ-GCCTTCAAGGTGGTAGCCC-3’
	Reverse: 5ʹ-AAGGTGAAACTCTTGCCTCGTC-3’
ALP	Forward:5ʹ-CCTCGTTGACACCTGGAAGAG-3ʹ
	Reverse:5ʹ-TTCCGTGCGGTTCCAGA-3’
GAPDH	Forward:5′-CTTTGGTATCGTGGAAGGACTC-3
	Reverse:5′-GTAGAGGCAGGGATGATGTTCT-3′
U6	Forward:5′-CGCTTCACGAATTTGCGT-3′
	Reverse:5′-CTCGCTTCG CAGCACA-3′

### Cell transfection

BMSCs at the exponential stage were used for transfection. Briefly, 1 × 10^6^ cells were cultured in 6-well plates with 2 mL complete medium for 24 h until 90% confluency was achieved. HOTAIR overexpression vector pcDNA-HOTAIR, pcDNA negative control (NC), shRNA (sh)-HOTAIR (sh-HOTAIR 1#,2#,3#), sh-NNMT, and sh-NC were acquired from System Biosciences (SBI, USA). antago miR-378g and antagomiR-NC were synthesized by GenePharma (Shanghai, China). Herein, 1000 ng pcDNA, 1000 ng shRNA, 100 nM antago miRNA were transfected into BMSCs using the Lipofectamine 2000 kit (Invitrogen) and cultured according to the manufacturer’s instructions [31].

### Western blot

Total protein extraction was performed using cell lysis buffer (Ambion), and protein quality was determined using a bicinchoninic acid (BCA) protein assay (Pierce, USA). Briefly, 20 μg of protein was denatured at 95°C for 10 min in 5% β-mercaptoethanol solution and 95% Laemmli sample buffer (Bio-Rad). After separation by 12% sodium dodecyl sulfate–polyacrylamide gel electrophoresis (SDS–PAGE), the proteins were transferred to nitrocellulose membranes (Schleicher & Schuell, Germany) and blocked for 1 h at 25°C using 0.5% skimmed milk. Thereafter, the membrane was incubated with ALP (ab229126; Abcam, USA), RUNX2 (ab236639; Abcam), and GAPDH (ab8245; Abcam) at 25°C for 1 h, followed by secondary antibodies (Bethyl, USA) at 25°C for 1 h. Membrane-bound antibodies were assessed by chemiluminescence after rinsing with PBS [32].

### Luciferase assay

The luciferase assay was performed as previously described [33]. Briefly, starBase was used to detect the binding sites of HOTAIR and miR-378g, and TargetScan was used to identify the binding sites of miR-378g and NNMT. Wild-type (WT) HOTAIR or NNMT containing the putative binding site of miR-378g was amplified and subcloned into the pmirGlo luciferase reporter vector (Ambion). The QuickChange XL Site‐Directed Mutagenesis Kit (Stratagene, USA) was used to obtain the mutant HOTAIR (HOTAIR-MUT), the mutant NNMT mutated at three different binding sites (NNMT-MUT1, NNMT-MUT2, NNMT-MUT3), and the mutant NNMT mutated at all three sites (co-MUT). agomiR-378g or agomiR-NC was transfected into BMSCs with wild-type HOTAIR/NNMT or mutant HOTAIR/NNMT using the Lipofectamine 2000 kit. The dual-luciferase reporter system (Promega) and Glomax20/20 luminometer fluorescence detector (Promega) were used to quantify luciferase activity after transfection for 48 h.

### Data statistics

Data are expressed as mean ± standard deviation. One-way analysis of variance (ANOVA) or Student’s t-test was used for significance analysis. Statistical analyses were performed using GraphPad Prism version 6. The correlation between HOTAIR, NNMT, and miR-378g in clinical samples was evaluated using Pearson’s analysis. Statistical significance was set at p < 0.05.

## Results

In this study, we hypothesized that the lncRNA, HOTAIR/miR-378g/NNMT, plays a biological role in OP and the osteogenic differentiation of BMSCs. HOTAIR, miR-378g, and NNMT were designated as genes of interest through bioinformatics analysis. Clinical monitoring and functional loss-gain analysis showed that HOTAIR, miR-378g, and NNMT are involved in BMSC osteogenic differentiation and are associated with OP. Mechanistic analysis showed that HOTAIR, as a ceRNA, upregulated NNMT via sponge miR-378g, and thus significantly inhibited BMSC osteogenic differentiation.

### HOTAIR/miR-378g/NNMT axis in osteoporosis

**Figure 1.**
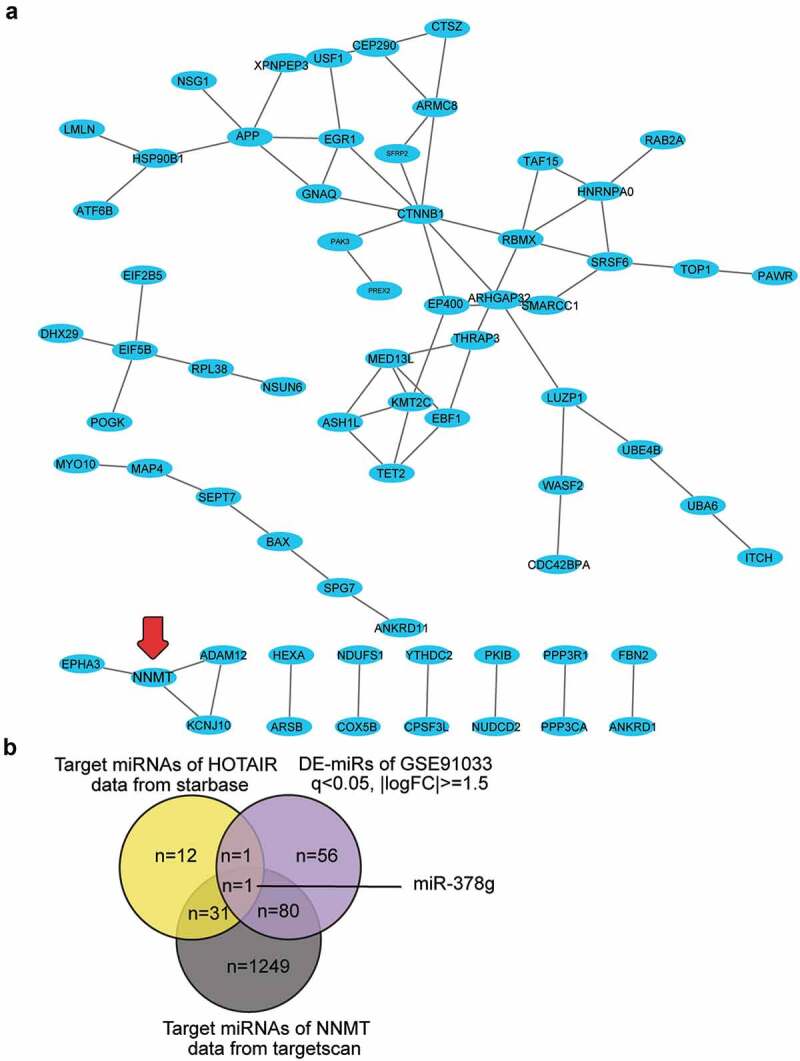
HOTAIR/miR-378g/NNMT axis may be involved in osteoporosis. (a) Protein–protein interaction analysis of the significantly upregulated genes of GSE35959 data series (adjusted P < 0.05 and logFC ≥ 2, n = 109) by string algorithm. (b) The intersection between the target miRNAs of HOTAIR, the target miRNAs of NNMT, and the differentially expressed miRNAs in osteoporosis (GSE91033 data). The target prediction of HOTAIR was conducted using the starBase algorithm, while that of NNMT was conducted using the TargetScan algorithm. The DE-miRNAs of GSE91033 were analyzed using the criteria of adjusted P < 0.05 and |logFC|≥1.5

HOTAIR has been reported to inhibit osteogenic differentiation, suggesting its osteoporosis-promoting effect [16]. However, the ceRNA mechanism involving HOTAIR has not been reported for osteoporosis. We uploaded the 109 differentially expressed genes in osteoporosis from GSE35959 to STRING 11.0 for protein-protein interaction analysis, and protein-protein interaction was visualized using Cytoscape. Sixty-six of the 109 genes were found to be closely associated. NNMT1 showed close interactions with KCNJ10, ADAM12, and EPHA3 (Figure 1(a)). NNMT1 was significantly upregulated in OVX-induced osteoporosis [26]; however, its role in osteoporosis has not been studied. By intersecting the target miRNAs of HOTAIR, the target miRNAs of NNMT, and the DE-miRNAs of GSE91033, we identified miR-378g as a potential miRNA that linked HOTAIR and NNMT in osteoporosis (Figure 1(b)).

### HOTAIR is highly expressed in OP and is associated with osteogenic differentiation

**Figure 2.**
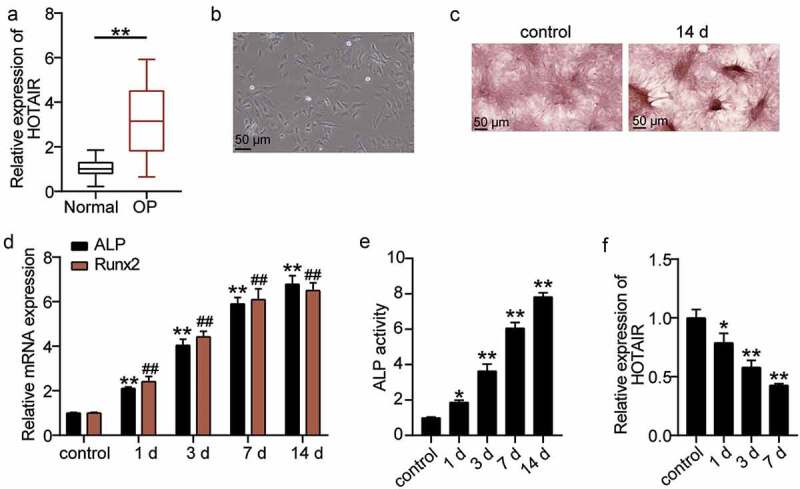
The relationship between HOTAIR expression level and osteogenesis. (a) HOTAIR expression levels in the serum of patients with non-osteoporotic bone fractures (normal) or osteoporosis (OP). **P < 0.001. (b) The shape of BMSCs on day 4 of osteogenic differentiation. (c) Following culture in osteogenic induction medium for 14 days, BMSCs exhibited more mineralized nodules according to Alizarin Red S staining. (d) The expression levels of the osteoblast marker genes, *ALP* and *RUNX2*, on different days of induction. **P < 0.001 vs. control in ALP. ##P < 0.001 vs. control in RUNX2. (e) ALP activity on different days of induction. **P < 0.001 vs. control. (f) The expression of HOTAIR on different days of induction. *P < 0.05, **P < 0.001 vs. control. BMSCs, Bone mesenchymal stem cells; HOTAIR, X–inactive specific transcript; ALP, alkaline phosphatase; RUNX2, runt related transcription factor 2. Data are expressed as mean ± standard error. In Panels D-F, one-way analysis of variance was used for data analysis, followed by Dunnett’s post hoc test. In Panel A, unpaired t-test was used for data analysis. The experiment was repeated three times

To explore the effect of HOTAIR on osteoporosis, qRT-PCR was used to determine HOTAIR expression in the sera of patients. HOTAIR expression in OP patients was found to increase by approximately threefold relative to that in normal patients (Figure 2(a)). Such finding indicates that HOTAIR may be related to OP. BMSCs were then used as the research object to induce osteogenic differentiation to observe the mechanism of HOTAIR. The morphology of cells was observed on day 4 after osteogenic differentiation. Accordingly, the cells were found to grow well and displayed spindle growth (Figure 2(b)). ARS staining is often used to detect osteoblast differentiation [34]. Therefore, ARS staining was used to observe the calcium nodules after osteogenic differentiation. Calcium deposition was found to be significant at 14 days after induction (Figure 2(c)). Runx2 is a key regulator of the osteoblast lineage and induces the maturation of osteoblast phenotypes [35]. ALP is a marker enzyme of mature osteoblasts, reflecting its osteogenic differentiation ability [36]. Hence, the expression levels of ALP and RUNX2, the key genes involved in osteogenic differentiation, were measured. The levels of ALP and RUNX2 increased with the time of osteogenic differentiation (Figure 2(d)). ALP activity was determined by ELISA, which confirmed that with the extension of osteogenic differentiation time, ALP activity increased (Figure 2(e)). Such finding indicated that BMSCs differentiated into osteoblasts. Furthermore, the level of HOTAIR expression during the osteogenic differentiation of BMSCs was measured, showing that HOTAIR expression decreased with differentiation time (figure 2(f)). Such finding suggests that HOTAIR is also involved in osteogenic differentiation.

### Downregulation of HOTAIR promotes the osteogenic differentiation of BMSCs, while upregulation of HOTAIR inhibits BMSC osteogenic differentiation

**Figure 3.**
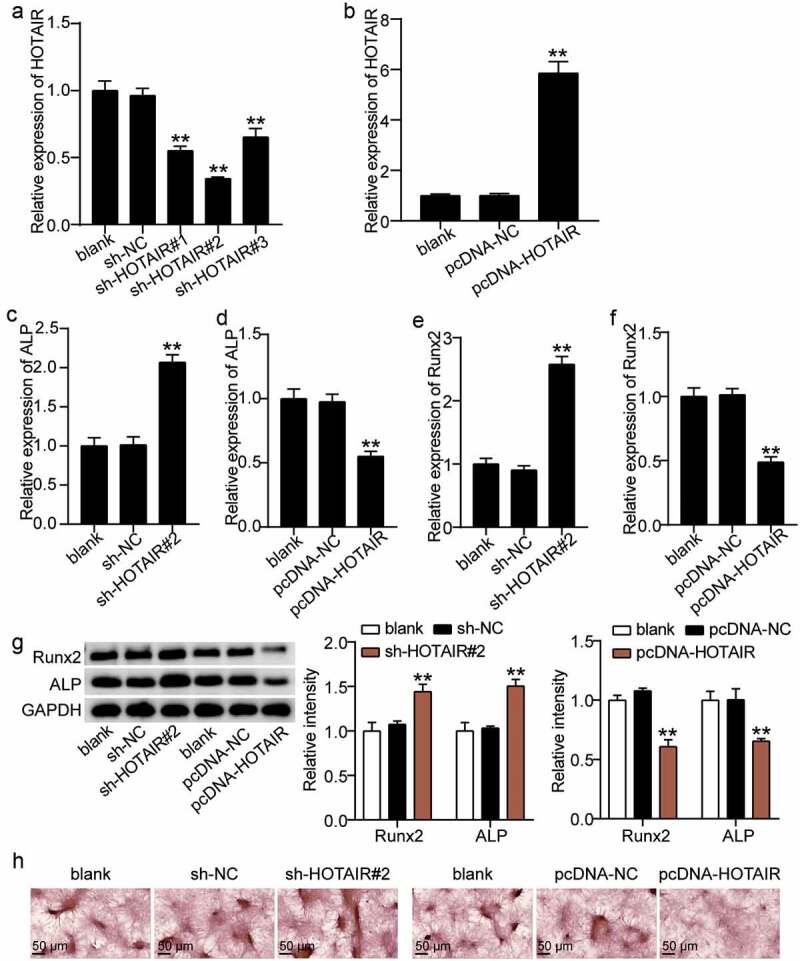
Effect of HOTAIR expression on osteogenesis. (a) HOTAIR expression in BMSCs treated with sh-NC, sh-HOTAIR 1#, sh-HOTAIR 2#, or sh-HOTAIR 3# detected by qRT-PCR. **P < 0.001 vs. sh-NC. (b) HOTAIR expression in BMSCs treated with pcDNA-NC or pcDNA-HOTAIR detected by qRT-PCR. **P < 0.001 vs. pcDNA-NC. (c) ALP expression in BMSCs treated with sh-NC or sh-HOTAIR 2# detected by qRT-PCR. (D) ALP expression in BMSCs treated with pcDNA-NC or pcDNA-HOTAIR detected by qRT-PCR. (e) RUNX2 expression in BMSCs treated with sh-NC or sh-HOTAIR 2# detected by qRT-PCR. (f) RUNX2 expression in BMSCs treated with pcDNA-NC or pcDNA-HOTAIR detected by qRT-PCR. (g) The protein expression of ALP and RUNX2 in BMSCs treated with sh-HOTAIR 2# or pcDNA-HOTAIR measured by Western blot analysis. (h) The mineralized nodules of BMSCs treated with sh-HOTAIR 2# or pcDNA-HOTAIR measured by Alizarin Red S staining. (c-g) **P < 0.001 vs. blank. BMSCs, Bone mesenchymal stem cells; HOTAIR, X–inactive specific transcript; ALP, alkaline phosphatase; RUNX2, runt related transcription factor 2. pcDNA-HOTAIR, HOTAIR overexpression vector; pcDNA-NC, negative control of HOTAIR overexpression vector; sh-HOTAIR, shRNA of HOTAIR; sh-NC, negative control of shRNA. Data are expressed as mean ± standard error. One-way analysis of variance was used for data analysis, followed by Dunnett’s post hoc test. The experiment was repeated three times

We attempted to elucidate the mode of action of HOTAIR on the osteogenic differentiation of BMSCs. Abnormal regulation of HOTAIR expression in BMSCs showed that the HOTAIR expression levels in the sh-HOTAIR 1#, sh-HOTAIR 2#, and sh-HOTAIR 3# groups were reduced by approximately 40%, 60%, and 30%, respectively, compared with the sh-NC group (Figure 3(a)). Such finding indicates that HOTAIR expression in BMSCs was successfully knocked down and sh-HOTAIR 2# was selected for follow-up experiments. After the overexpression of HOTAIR, the expression level of HOTAIR in BMSCs increased by approximately sixfold (Figure 3(b)). Thus, we proceeded to explore the effect of changes in HOTAIR on ALP and RUNX2. ALP and RUNX2 expression levels were found to increase by 2.0- and 2.5-fold, respectively after silencing HOTAIR, and decrease by 45% and 50% after increasing HOTAIR (Figure 3(c–f)). In addition, Western blot analysis showed that the RUNX2 and ALP expression of sh-HOTAIR 2# was 1.4- and 1.6-fold higher than that of sh-NC, respectively, while the overexpression of HOTAIR reduced the expression levels of ALP and RUNX2 to 40% and 35% of the pcDNA-NC group, respectively (Figure 3(g)). ARS staining also showed that calcium deposition increased after the transfection of sh-HOTAIR 2# into BMSCs but was inhibited after BMSCs were transfected with pcDNA-HOTAIR (Figure 3(h)). Such finding suggests that HOTAIR suppresses the osteogenic differentiation of BMSCs.

### HOTAIR targets miR-378g

**Figure 4.**
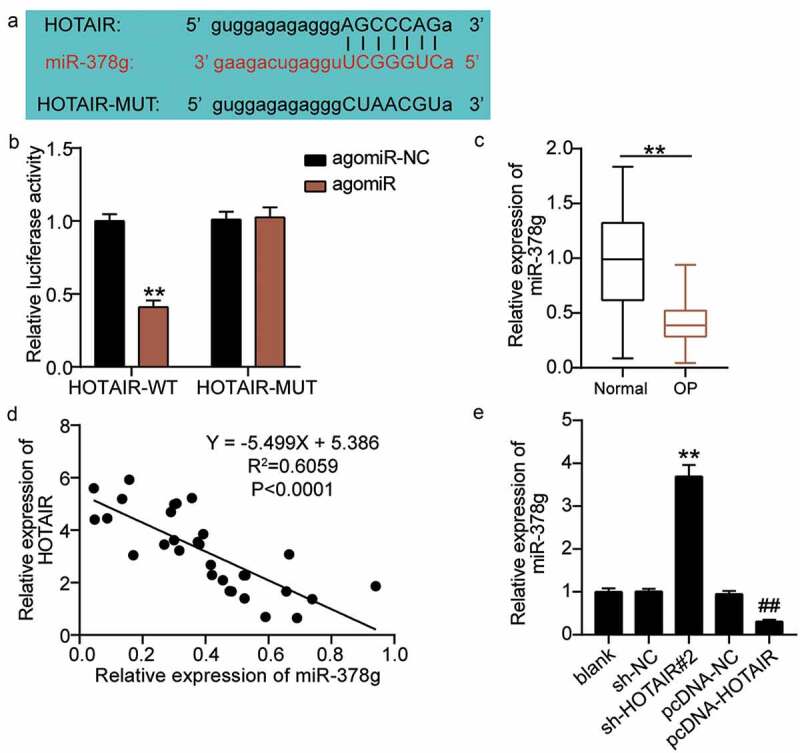
HOTAIR targets miR-378g. (a) Prediction of the binding sites between HOTAIR and miR-378g by starBase. (b) Binding between HOTAIR and miR-378g as assessed by the luciferase activity assay. **P < 0.001 vs. agomiR-NC. (c) miR-378g expression levels in the serum of patients with non-osteoporotic bone fractures (normal) or osteoporosis (OP). **P < 0.001. (d) The correlation between HOTAIR and miR-378g analyzed by Pearson’s analysis. (e) The miR-378g expression in BMSCs treated with sh-NC or sh-HOTAIR 2# detected by qRT-PCR. **P < 0.001 vs. sh-NC, ##P < 0.001 vs. pcDNA-NC. WT, wild-type; MUT, mutant type; HOTAIR, X–inactive specific transcript; miR-378g, microRNA-378 g; sh-HOTAIR, shRNA of HOTAIR; sh-NC, negative control of shRNA; pcDNA-NC, negative control of HOTAIR overexpression; pcDNA-HOTAIR, HOTAIR overexpression vectors. Data are expressed as mean ± standard error. In Panels B and E, one-way analysis of variance was used for data analysis, followed by Tukey’s post hoc test. In Panel C, unpaired t-test was used for data analysis. The experiment was repeated three times

As the effect of HOTAIR on osteogenic differentiation is well known, we expect to elucidate the mechanism of action of HOTAIR. The starBase sharing site revealed the existence of targeting sites for HOTAIR and miR-378g (Figure 4(a)). By using a dual-luciferase assay to verify the results, the luciferase activity was found to decrease after the co-transfection of wild-type HOTAIR and agomiR-378g; however, no significant change was found after the co-transfection of mutant HOTAIR and agomiR-378g (Figure 4(b)). These results proved that HOTAIR targets miR-378g. qRT-PCR was employed to determine serum miR-378g levels. Based on the results, miR-378g levels in osteoporotic patients decreased by nearly 70% compared with those in non-osteoporotic patients (Figure 4(c)). Pearson analysis revealed a negative correlation between HOTAIR and miR-378g expression levels in the serum of OP patients (Figure 4(d)). Additionally, qRT-PCR analysis showed that the expression level of miR-378g increased by approximately 3.7-fold after the downregulation of HOTAIR, and decreased by approximately 70% after the upregulation of HOTAIR (Figure 4(e)), ultimately revealing that HOTAIR targets and negatively regulates miR-378g expression.

### HOTAIR restrains osteogenic differentiation by inhibiting miR-378g

**Figure 5.**
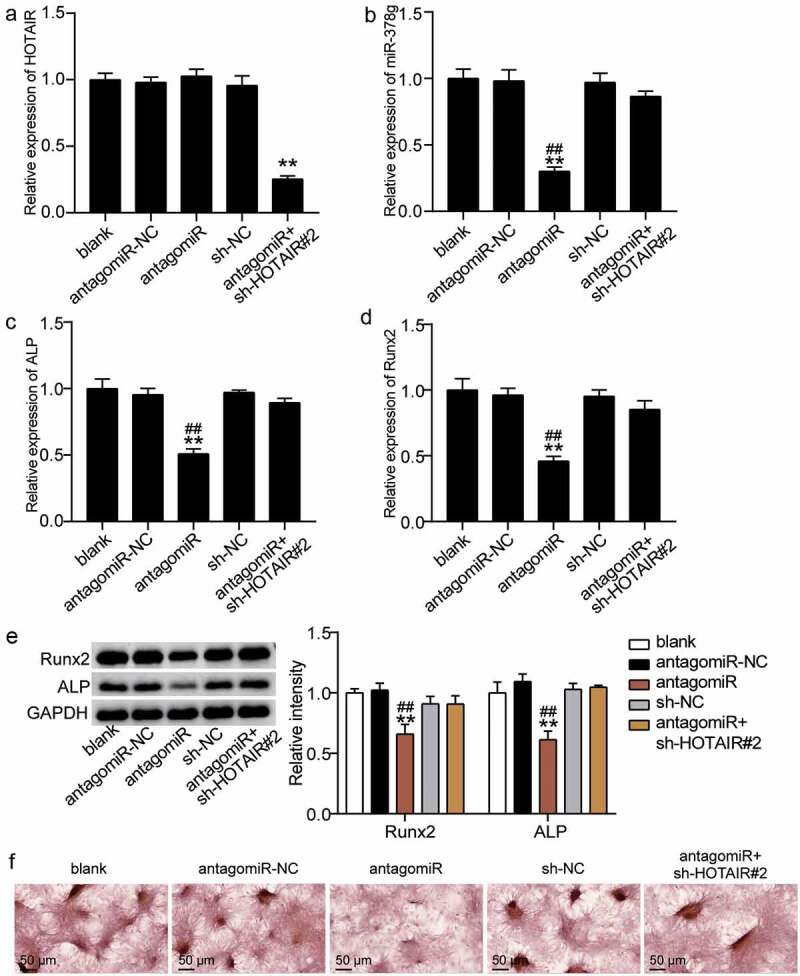
HOTAIR suppresses osteogenesis by inhibiting miR-378g. (a) HOTAIR expression in BMSCs treated with sh-NC, sh-HOTAIR 2#, antagomiR-NC, or antagomiR. (b) miR-378g expression in BMSCs treated with sh-NC, sh-HOTAIR 2#, antagomiR-NC, or antagomiR. (c) ALP expression in BMSCs treated with sh-NC, sh-HOTAIR 2#, antagomiR-NC, or antagomiR. (d) RUNX2 expression in BMSCs treated with sh-NC, sh-HOTAIR 2#, antagomiR-NC, or antagomiR. (e) ALP and RUNX2 protein expression in BMSCs treated with sh-NC, sh-HOTAIR 2#, antagomiR-NC, or antagomiR. (f) The mineralized nodules of BMSCs treated with sh-NC, sh-HOTAIR 2#, antagomiR-NC, or antagomiR measured by Alizarin Red S staining. **P < 0.001 vs. blank. ##P < 0.001 vs. sh-HOTAIR#2+ antagomiR. BMSCs, Bone mesenchymal stem cells; HOTAIR, X–inactive specific transcript; ALP, alkaline phosphatase; RUNX2, runt related transcription factor 2. sh-HOTAIR, shRNA of HOTAIR; sh-NC, shRNA of negative control; antagomiR, antago miR-378g; antagomiR-NC antago miR-378g negative control. Data are expressed as mean ± standard error. One-way analysis of variance was used for data analysis, followed by Tukey’s post hoc test. The experiment was repeated three times

We examined the regulatory mechanisms of HOTAIR and miR-378g on osteogenic differentiation. When antago miR-378g was transfected into BMSCs, the HOTAIR level did not change, while the expression level of miR-378g decreased by 70% (Figure 5(a,b)). However, the HOTAIR level was reduced by 75% compared to that of the blank group after the co-downregulation of HOTAIR and miR-378g, while the miR-378g level was similar to that of the blank group (Figure 5(a,b)). Such finding suggests that HOTAIR and miR-378g are co-downregulated. Furthermore, the ALP and RUNX2 levels were found to decrease by approximately 50% and 55%, respectively, after knockdown of miR-378g compared with antagomiR-NC, and the low expression of HOTAIR reduced the inhibitory effect of miR-378g knockdown on ALP and RUNX2 (Figure 5(c,d)). Similarly, Western blotting showed that the levels of ALP and RUNX2 decreased after miR-378g interference, while the co-knockdown of HOTAIR and miR-378g showed no significant changes in the protein levels of ALP and RUNX2 (Figure 5(e)). In addition, ARS staining showed that calcium deposition decreased after transfection with antago miR-378g, and this suppression effect was reversed by knockdown of HOTAIR (figure 5(f)). These findings indicate that the inhibitory effect of HOTAIR on osteogenic differentiation may be mediated via miR-378g.

### miR-378g targets NNMT

**Figure 6.**
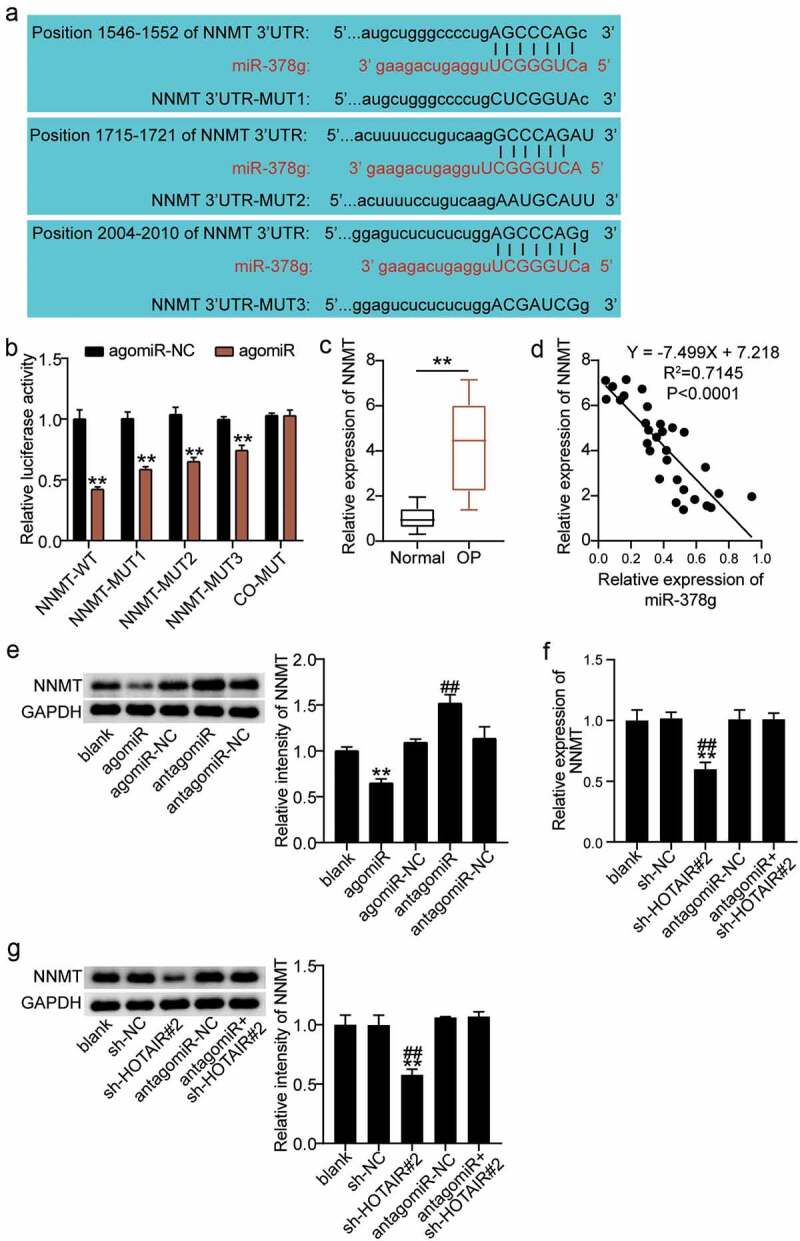
miR-378g targets NNMT mRNA. (a) Prediction of the binding sites between NNMT and miR-378g by TargetScan. (b) The binding between NNMT and miR-378g as assessed by the luciferase activity assay. **P < 0.001 vs. agomiR-NC. (c) NNMT mRNA expression levels in the serum of patients with non-osteoporotic bone fractures (normal) or osteoporosis (OP). **P < 0.001. (d) The correlation between NNMT and miR-378g assessed using Pearson’s analysis. (e) NNMT protein expression in BMSCs treated with agomiR-NC, agomiR, antagomiR-NC, or antagomiR. **P < 0.001 vs. agomiR-NC. ##P < 0.001 vs. antagomiR-NC. (f) NNMT expression in BMSCs treated with sh-NC, sh-HOTAIR 2#, antagomiR-NC, or antagomiR. (g) NNMT protein expression in BMSCs treated with sh-NC, sh-HOTAIR 2#, antagomiR-NC, or antagomiR. (f-g) **P < 0.001 vs. sh-NC. ##P < 0.001 vs. sh-HOTAIR#2+ antagomiR. BMSCs, Bone mesenchymal stem cells; HOTAIR, X–inactive specific transcript; NNMT, Nicotinamide N-methyltransferase. sh-HOTAIR, shRNA of HOTAIR; sh-NC, shRNA of negative control; antagomiR, antago miR-378g; antagomiR-NC antago miR-378g negative control. Data are expressed as mean ± standard error. One-way analysis of variance was used for data analysis, followed by Tukey’s post hoc test. The experiment was repeated three times

miR-378g participates in the regulation of various physiological processes through the negative regulation of its target gene [37]. We proceeded to investigate the downstream target genes of miR-378g. As shown in Figure 6(a), NNMT contains three targets associated with miR-378g. A dual-luciferase assay revealed that compared with agomiR-NC, luciferase activity decreased by 65% after co-transfection of WT and agomiR, and decreased by 55%, 40%, and 30%, respectively, after co-transfection of MUT1, MUT 2, or MUT3, and agomiR. In addition, no significant change in luciferase activity was found after co-transfection with co-MUT and agomiR (Figure 6(b)), revealing miR-378g targeting combined with the NNMT 3ʹUTR. Moreover, serum monitoring showed that the expression of NNMT in the OP group was significantly upregulated compared to that in the normal group (Figure 6(c)). In addition, Pearson analysis showed that serum NNMT was negatively correlated with the expression of miR-378g in OP patients (Figure 6(d)). Furthermore, the regulatory effects of miR-378g on NNMT were monitored. Western blotting showed that NNMT decreased after miR-378g overexpression, but increased after miR-378g interference (Figure 6(e)). In addition, NNMT mRNA and protein levels decreased in the sh-HOTAIR 2# group compared to those in the sh-NC group or antagomiR + sh-HOTAIR 2# group (Figure 6(f,g)). Such finding indicates that miR-378g negatively regulates NNMT, while HOTAIR reverses this regulatory effect.

### miR-378g promotes osteogenic differentiation by inhibiting NNMT

**Figure 7.**
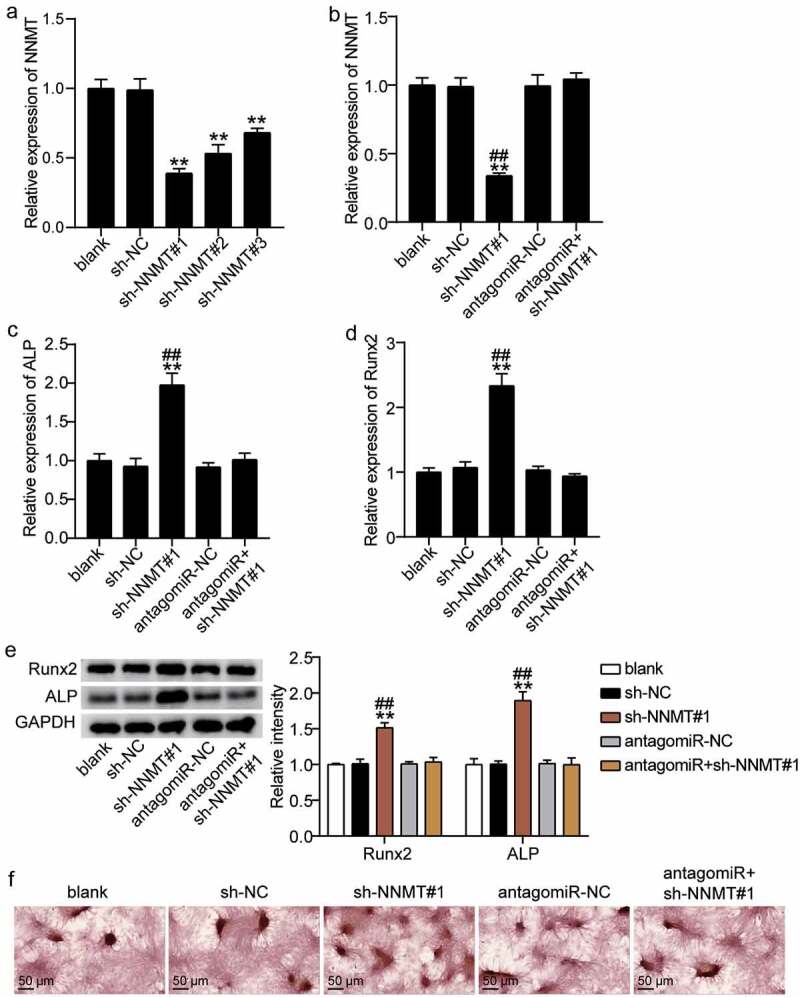
miR-378g enhances osteogenesis by inhibiting NNMT mRNA. (a)NNMT expression in BMSCs treated with sh-NC, sh-NNMT 1#, sh-NNMT 2#, or sh-NNMT 3#. **P < 0.001 vs. sh-NC. (b) NNMT expression in BMSCs treated with sh-NC, sh-NNMT 1#, antagomiR-NC, or antagomiR. (c) ALP expression in BMSCs treated with sh-NC, sh-NNMT 1#, antagomiR-NC, or antagomiR. (d) RUNX2 expression in BMSCs treated with sh-NC, sh-NNMT 1#, antagomiR-NC, or antagomiR. (e) ALP and RUNX2 protein expression in BMSCs treated with sh-NC, sh-NNMT 1#, antagomiR-NC, or antagomiR. (b-e) **P < 0.001 vs. blank. ##P < 0.001 vs. sh-NNMT#1+ antagomiR. (f) The mineralized nodules of BMSCs treated with sh-NC, sh-NNMT 1#, antagomiR-NC, or antagomiR measured by Alizarin Red S staining. BMSCs, Bone mesenchymal stem cells; ALP, alkaline phosphatase; RUNX2, runt related transcription factor 2; NNMT, Nicotinamide N-methyltransferase. sh-NNMT, shRNA of NNMT; sh-NC, shRNA of negative control; antagomiR, antago miR-378g; antagomiR-NC antago miR-378g negative control. Data are expressed as mean ± standard error. One-way analysis of variance was used for data analysis, followed by Tukey’s post hoc test. The experiment was repeated three times

NNMT and miR-378g of BMSCs were downregulated to explore the changes in osteogenic differentiation. NNMT knockdown was transferred to BMSCs with either sh-NNMT #1, sh-NNMT T #2, or sh-NNMT #3, and the level of NNMT was found to decrease by approximately 60%, 50%, and 35%, respectively (Figure 7(a)). Sh-NNMT #1 was selected for the follow-up experiments. qRT-PCR showed that miR-378g reversed the effect of NNMT knockdown on NNMT levels (Figure 7(b)). The effect of NNMT on the mRNA expression levels of ALP and RUNX2 in BMSCs was further studied. ALP and RUNX2 mRNA levels in the sh-NNMT #1 group were 1.8-fold and 2.4-fold higher than levels in the sh-NC group. Further, antago miR-378g partially eliminated the effect of sh-NNMT #1 on ALP and RUNX2 (Figure 7(c,d)). Based on Western blotting, ALP and RUNX2 levels increased by 1.5-fold and 2.0-fold after NNMT knockdown, and were similar to the blank group after knockdown of NNMT and miR-378g (Figure 7(e)). Additionally, ARS staining showed that the inhibition of NNMT expression increased the degree of calcium nodules, while miR-378g silencing reversed the effect of NNMT knockdown on calcium nodules (figure 7(f)). These results indicate that miR-378g promotes osteogenic differentiation by targeting NNMT.

## Discussion

Dysregulation of osteoblast differentiation is associated with the progression of osteoporosis. Further, ALP, calcium deposition, and RUNX2 levels are often employed to illustrate osteoblast differentiation and its potential for treating OP [38]. In this study, we elucidated the effect of HOTAIR/miR-378g/NNMT on the osteogenic differentiation of BMSCs based on changes in ALP, RUNX2, and calcium deposition levels, thereby providing a potential biomarker for the treatment of OP.

HOTAIR is closely related to bone formation. Zhan et al. [39] found that the overexpression of HOTAIR induced extracellular matrix degradation, apoptosis, and senescence in nucleus pulposus cells. Misawa et al. [40] reported that the downregulation of HOTAIR resulted in the upregulation and increased mineralization of osteosarcoma cells and ALP expression in mineralized medium. In this study, we found that HOTAIR was overexpressed in the serum of OP patients and was underexpressed during the osteogenic differentiation of BMSCs, which align with the findings of Shen et al. [16]. In addition, interference with HOTAIR enhanced the expression of ALP and RUNX2 and promoted calcium deposition in BMSCs. This finding was similar to that observed in osteosarcoma cells by Misawa et al. [40]. Furthermore, this study revealed that HOTAIR upregulation inhibits the osteogenic differentiation of BMSCs, which suggests that HOTAIR may be involved in BMSC-mediated OP.

LncRNAs may act as competing endogenous RNAs (ceRNAs) by competitively binding to miRNAs through their miRNA response elements, thereby regulating the expression levels of miRNAs that target mRNAs [41]. Thus, lncRNAs can act as miRNA sponges, reducing their regulatory effects on mRNAs [42]. Abnormal lncRNA-miRNA-mRNA networks of ceRNAs have been reported to be involved in the development of OP [43]. For example, silencing LNC_000052 inhibited PIK3R1 expression by upregulating miR-96-5p to promote the proliferation, migration, and osteogenesis of osteoporotic BMSCs and inhibit cell apoptosis [14]. The lncRNA, NEAT1, regulates the expression of OP-related genes (*ALP, OCN*, and *OPN*) in BMSCs by regulating the miR-29b-3p-BMP1 axis [44]. miR-378 has been shown to promote BMSC differentiation into osteoclasts in vitro [45]. In addition, another study revealed that miR-378 was underexpressed in high glucose-induced OP mice; however, miR-378 increased ALP activity and promoted RUNX2 expression [46]. This study found that miR-378g expression in the serum of OP patients was downregulated, interference miR-378g inhibited ALP and RUNX2 levels, and reduced calcium deposition, which is similar to the findings of previous studies. The molecular mechanism revealed that miR-378g was negatively regulated by HOTAIR, and interfering with miR-378g eliminated downregulated HOTAIR-induced osteogenesis. HOTAIR exacerbates bone marrow mesenchymal stem cell-mediated osteoporosis through sponging miR-378g.

Accumulating literature suggests that miR-378g can bind to different mRNAs and participate in various physiological processes. For example, Liu et al. [47] reported that miR-378g targeted CHI3L1 to regulate the migration, invasion, and EMT of ovarian cancer cells. Li et al. [48] showed that *HOXC13*, a target gene of miR-378g, accelerates the malignant behavior of oral squamous cell carcinoma cells. Lin et al. [37] revealed that miR-378g partially enhances the radiosensitivity of NPC cells by targeting SHP-1. In this study, miR-378g was found to be a potential miRNA upstream of NNMT by bioinformatics analysis. Moreover, targeting analysis demonstrated that NNMT has a binding site for miR-378g, is a target gene for miR-378g, and is positively regulated by HOTAIR. Interference with NNMT promoted the osteogenic differentiation of BMSCs and reversed the inhibitory effect of miR-378g knockdown on osteogenic differentiation. This finding is consistent with that of Wang et al. [26]. HOTAIR acts as a ceRNA to damage the osteogenic differentiation of BMSCs through the miR-378g/NNMT axis.

This study had some limitations. First, miR-378g/NNMT is not the only regulatory pathway for HOTAIR, and other mechanisms by which HOTAIR affects OP need to be elucidated. In addition, the effect of HOTAIR on OP needs to be further demonstrated at the animal level.

## Conclusion

In summary, HOTAIR was found to be overexpressed in OP. Further downregulation of the ceRNA, NNMT, by miR-378g sponge significantly inhibited the osteogenic differentiation of BMSCs. Therefore, our findings demonstrate that HOTAIR/miR-378g/NNMT is a potential target for the osteogenic differentiation of BMSCs and has a certain value in the diagnosis and treatment of OP.

## Data Availability

The datasets used and/or analyzed during the current study are available from the corresponding author on reasonable request.

## Highlights

- HOTAIR and NNMT are highly expressed in OP and inhibit BMSC osteogenic differentiation.
- miR-378g alleviates OP by inducing the osteogenic differentiation of BMSCs.
- HOTAIR acts as a ceRNA to damage BMSC osteogenic differentiation via the miR-378g/NNMT axis.
